# Outcome of diagnostic intervention predicts health-related quality of life scores among children with food allergy

**DOI:** 10.1186/1710-1492-8-S1-A9

**Published:** 2012-11-02

**Authors:** Linda Kirste, Tim K Takaro, Boris Kuzeljevic, Tiffany Wong, Edmond S Chan

**Affiliations:** 1 Faculty of Health Sciences, Simon Fraser University, Burnaby, BC, Canada, V5A 1S6; 2 Child and Family Research Institute, Vancouver, BC, Canada, V5Z 4H4; 3 Department of Pediatrics, Division of Allergy, University of British Columbia, Vancouver, BC, Canada, V6H 3V4

## Background

Access to diagnostic care, regardless of diagnostic outcome, may attenuate the negative impact of food allergy on health-related quality of life (HRQL). We sought to determine if improved HRQL can be demonstrated among children, 0-12 years, who receive diagnostic care for food allergy in an allergy clinic setting.

## Methods

Parents attending clinic with their child completed the Food Allergy Quality of Life Questionnaire Parent Form before and after their visit. Parents with children on the clinic waitlist served as controls. HRQL scores were analyzed according to visit outcome: fewer or same number of food allergies. A sub-analysis of scores among children who underwent an oral food challenge (OFC) was conducted. The General Linear Model for Repeated Measures was used to compare changes in score over time between outcomes, and to test for interaction between score changes and outcomes.

## Results

Mean pre-/post-visit scores were 1.93/1.68 for fewer (n = 64), 2.37/2.37 for same (n = 36), and 1.70/1.79 for controls (n = 59). Interaction between score change and visit outcome was significant (F 3.355, p = 0.037). Pre-/post-visit scores for OFC outcomes only were 2.24/2.03 for fewer (n = 35) and 2.03/2.53 for same (n = 10) number of food allergies. Interaction between score change and OFC outcome was significant (F 5.518, p = 0.023).

## Conclusions

Improvement in HRQL associated with food allergy diagnostic care appears to be dependent on visit outcome. Diagnosis of fewer food allergies predicted improvement in HRQL scores among children; this improvement may be most pronounced among those who receive oral food challenges.

